# Antibiotic Effects on Microbial Communities Responsible for Denitrification and N_2_O Production in Grassland Soils

**DOI:** 10.3389/fmicb.2018.02121

**Published:** 2018-09-11

**Authors:** Miguel Semedo, Bongkeun Song, Tavis Sparrer, Rebecca L. Phillips

**Affiliations:** ^1^Department of Biological Sciences, Virginia Institute of Marine Science, College of William & Mary, Gloucester Point, VA, United States; ^2^Ecological Insights Corporation, Hazelton, ND, United States

**Keywords:** denitrification, nitrous oxide, tetracycline, *nosZ*, bacteria, fungi

## Abstract

Antibiotics in soils may affect the structure and function of microbial communities. In this study, we investigated the acute effects of tetracycline on soil microbial community composition and production of nitrous oxide (N_2_O) and dinitrogen (N_2_) as the end-products of denitrification. Grassland soils were pre-incubated with and without tetracycline for 1-week prior to measurements of N_2_O and N_2_ production in soil slurries along with the analysis of prokaryotic and fungal communities by quantitative polymerase chain reaction (qPCR) and next-generation sequencing. Abundance and taxonomic composition of bacteria carrying two genotypes of N_2_O reductase genes (*nosZ*-I and *nosZ*-II) were evaluated through qPCR and metabolic inference. Soil samples treated with tetracycline generated 12 times more N_2_O, but N_2_ production was reduced by 84% compared to the control. In parallel with greater N_2_O production, we observed an increase in the fungi:bacteria ratio and a significant decrease in the abundance of *nosZ*-II carrying bacteria; *nosZ*-I abundance was not affected. *NosZ*-II-carrying *Bacillus* spp. (Firmicutes) and *Anaeromyxobacter* spp. (Deltaproteobacteria) were particularly susceptible to tetracycline and may serve as a crucial N_2_O sink in grassland soils. Our study indicates that the introduction of antibiotics to agroecosystems may promote higher N_2_O production due to the inhibitory effects on *nosZ*-II-carrying communities.

## Introduction

Microbial denitrification is a dominant respiratory pathway for reactive N-removal in terrestrial and aquatic ecosystems. Diverse microorganisms belonging to several genera of bacteria, archaea, and fungi perform denitrification (Stein and Klotz, 2017). Dinitrogen gas (N_2_) is the end-product of complete denitrification: the stepwise reduction of nitrate (${\text{NO}}_{3}^{-}$) and nitrite (${\text{NO}}_{2}^{-}$) to nitric oxide (NO), nitrous oxide (N_2_O), and dinitrogen (N_2_). However, many microorganisms do not carry the necessary genes to perform complete denitrification and may instead release N_2_O as the end-product of incomplete denitrification. Since N_2_ is radiatively inert and N_2_O is a potent greenhouse gas and the dominant source of stratospheric ozone depletion, the atmospheric impacts of complete and incomplete denitrification are dramatically different (Ravishankara et al., 2009; Neubauer and Megonigal, 2015). Denitrifiers capable of complete denitrification to N_2_ rely on the presence and expression of the *nosZ* gene, which encodes N_2_O reductase, the enzyme that converts N_2_O to N_2_ (Thomson et al., 2012). *NosZ*-carrying prokaryotes, found in both Archaea and Bacteria, consume N_2_O and therefore are an important biological sink for N_2_O in soils (Ma et al., 2011; Jones et al., 2014; Domeignoz-Horta et al., 2015; Samad et al., 2016). Because the *nosZ* gene is missing in fungal genomes, fungal denitrifiers have an incomplete denitrification pathway and are a source of N_2_O (Shoun et al., 2012; Maeda et al., 2015; Mothapo et al., 2015).

Recent studies of *nosZ* gene diversity have revealed the presence of two *nosZ* genotypes, clade I (*nosZ*-I) and clade II (*nosZ*-II), that have different phylogenetic, physiologic, and ecological properties (Hallin et al., 2017). The taxonomic and genetic diversity of *nosZ*-II carrying prokaryotes is greater than *nosZ*-I and is positively correlated to soil N_2_O sink capacity (Jones et al., 2013, 2014; Domeignoz-Horta et al., 2015). Additionally, a larger percent of *nosZ*-II carrying bacteria do not possess the genes that encode for nitrite reductase (Nir) and nitric oxide reductase (Nor) enzymes, which are necessary to produce N_2_O (Graf et al., 2014; Jones et al., 2014). This indicates that *nosZ-II* carrying bacteria are mostly N_2_O consumers, rather than N_2_O producers. The abundance and diversity of the two clades also appear to respond differently to environmental parameters including pH, moisture, and nutrient concentrations (Jones et al., 2014; Domeignoz-Horta et al., 2015; Samad et al., 2016). When considering responses to environmental contaminants, such as antibiotics, the differential responses of the two *nosZ* genotypes have not been studied yet.

Antibiotics are introduced into soils from multiple anthropogenic sources, such as in applications of animal manure and biosolids, inappropriate disposal of unused medicines, and in wastewater treatment effluents (Boxall, 2004). Animals excrete between 17 and 90% of antibiotics administered during livestock production in their feces and urine (Massé et al., 2014), and soil concentrations can range from a few microgram to gram per kilogram of soil (Thiele-Bruhn, 2003). Animal manures with antibiotics are frequently applied to agricultural soils as fertilizer (Sengeløv et al., 2003; Zhu et al., 2013) where they can affect denitrification activity of the microbial communities (Kotzerke et al., 2008; DeVries et al., 2015; Sun et al., 2017). Various studies have shown that antibiotics can decrease the abundance and activity of bacterial denitrifiers in soils, coastal sediments, and groundwater (Costanzo et al., 2005; Kotzerke et al., 2008; Kleineidam et al., 2010; Underwood et al., 2011; Hou et al., 2015). For example, chlortetracycline and oxytetracycline were shown to inhibit denitrification activities in groundwater and estuarine sediments, respectively (Ahmad et al., 2014; Yin et al., 2017).

Antibiotics may also alter soil microbial community composition, with cascading effects on net N_2_O production. For example, the fungi:bacteria ratio may be greater following antibiotic exposure if bacteria are selectively inhibited by the antibiotic (Thiele-Bruhn and Beck, 2005; Hammesfahr et al., 2008; Demoling et al., 2009; Gutiérrez et al., 2010). Since fungal denitrifiers produce N_2_O while some bacterial denitrifiers can reduce N_2_O to N_2_, an increase in the fungi:bacteria ratio is expected to increase N_2_O production from denitrification. However, that will also depend on which bacterial denitrifiers are inhibited. A selective inhibition of denitrifying bacteria carrying the *nosZ* gene would lower the N_2_O sink capacity (DeVries et al., 2015; Hou et al., 2015; Wu et al., 2017; Yin et al., 2017), leading to increased N_2_O production and decreased N_2_ production. Alternatively, all denitrifying bacteria (those with and without the *nosZ* gene) could be inhibited by antibiotics, leading to an overall decrease in both N_2_O and N_2_ production (Costanzo et al., 2005; Kotzerke et al., 2008; Conkle and White, 2012; Sun et al., 2017). Elucidating how antibiotics might alter microbial communities, including the two clades of *nosZ*-carrying prokaryotes and fungi, is therefore fundamental to understanding the effects on net N_2_O production from denitrification.

The objective of this research was to investigate the microbial community changes associated with the antibiotic impacts on microbial denitrification and N_2_O production. To achieve this goal, we conducted a laboratory experiment with grassland soil samples treated with tetracycline, an inhibitor of bacterial protein synthesis that belongs to one of the largest classes (tetracyclines) of antimicrobials used in the United States livestock industry (USEPA, 2013). Tetracycline compounds are ranked second in production and usage of antibiotics worldwide (Daghrir and Drogui, 2013). We hypothesized that tetracycline would cause a shift in microbial community structure, leading to a greater relative abundance of fungi to bacteria, lower abundance of *nosZ*-carrying bacteria, lower N_2_ production, and greater net production of N_2_O. This is the first report simultaneously evaluating the impacts of antibiotic exposure on prokaryotic and fungal communities as well as the two clades of *nosZ*-carrying bacteria associated with soil N_2_O emission.

## Materials and Methods

### Soil Collection

Soils were collected from a managed grassland farm (60 ha) in Emmons County, ND, United States (46°24′22^′′^; 100°23′16^′′^), where there had been no history of antibiotic or other agro-chemical application (including fertilizer), based on 80 years of farm records. Since 1990, the farm has been enrolled in the Conservation Reserve Program (CRP) because the soils were classified as highly erodible and not suitable for crop production by the United States Department of Agriculture Farm Services Agency (USDA-FSA). The CRP allows only limited grassland harvest (every 3–5 years) and no grazing (Phillips et al., 2015). A total of 16 plots (1 m^2^) were established within a 0.2 ha area to collect soil core samples following spring thaw on March 19, 2014. Air temperature at the time of coring was 2°C.

Two sets of surface soil samples (2.5 dia. × 10 cm depth) were collected using a hand auger at random locations within each plot and stored at 4°C. One set was collected to determine background bulk density, gravimetric soil moisture, total organic carbon (TOC), total nitrogen (TN), pH, and soil texture (sand, silt, and clay). Porosity and percent water-filled pore space (%WFPS) at the time of soil collection were calculated based on bulk density and moisture using a particle size density of 2.65 g cm^-3^. Soil sampling and measurement protocols are detailed in Phillips et al. (2012). The second set of soil samples were collected for tetracycline exposure incubations (see below). Before coring each plot, the auger was carefully cleaned and rinsed with alcohol. Three small diameter cores were collected per plot and mixed together to form one composited sample per plot. These were immediately shipped to the Virginia Institute of Marine Science (VIMS) for the antibiotic exposure work.

### Exposure of Soil to Tetracycline

Each of the 16 soil samples was split into two groups and 15 g of soil were amended with either 7.5 ml of autoclaved DI water (control group) or tetracycline (tetracycline group) delivered in 7.5 ml of autoclaved DI water (final tetracycline concentration of 1 mg g^-1^ soil). The tetracycline concentration used here is within the range of antibiotic concentrations known to repress microbial iron (III) reduction and denitrification (Thiele-Bruhn, 2005; Long et al., 2013). Each paired sample, control and tetracycline, was incubated in the dark at room temperature for 1 week. After incubation, samples were split into two groups, one for N_2_O and N_2_ production rate measurements, and one, which was stored at -80°C, for microbial community analysis and soil properties measurements.

### Soil Properties of Incubated Samples

Soil pH was determined using a glass electrode with a soil:deionized water mixture of 1:1 (w/v). Percent organics was determined by loss of ignition (500°C, 4 h). TOC and TN were quantified using the Exeter CHN model 440 CE analyzer. Soil ${\text{NO}}_{3}^{-}$ and ${\text{NO}}_{4}^{+}$ were measured in a Lachat QC8000 FIA after extraction with 2 M KCl (2:1 KCl to sediment ratio).

### Activity Measurements: N_2_O and N_2_ Production Rates

All 16 pairs of samples were used for soil slurry incubation experiments to measure potential rates of denitrification (moles of added ^15^N-labeled ${\text{NO}}_{3}^{-}$ transformed to ^30^N_2_) as well as total N_2_O production, following the method described by Long et al. (2013). One gram of each soil sample was pre-incubated anaerobically, after flushing with He gas, in 12-ml Exetainer tubes overnight. Two sets of tubes were prepared to measure N_2_ and N_2_O production from each sample. Pre-incubation served to deplete the resident soil ${\text{NO}}_{3}^{-}$ and ${\text{NO}}_{2}^{-}$ pools (NOx) prior to spiking with 200 nmol of potassium nitrate (K^15^NO_3_: 99%). Before the addition of potassium nitrate, all tubes were reflushed with He for 5 min to remove background N_2_, CO_2_, and other atmospheric gases. Time course incubations (time points 0 and 1 h after ^15^${\text{NO}}_{3}^{-}$ spike) were carried out in duplicate at room temperature. A 0.2 ml of potassium hydroxide (KOH) solution (4 M) was added at each time point to stop microbial activity. The ^30^N_2_ gas in the headspace was measured on a continuous-flow isotope ratio mass spectrometer (Thermo Delta V Advantage, Thermo Scientific) in line with an automated gas bench interface (Thermo Finnigan GasBench II, Thermo Scientific). The N_2_O gas in the headspace was measured using a gas chromatograph fitted with an electron capture detector (Shimadzu). The gas chromatograph was calibrated with commercial N_2_O standards and the coefficient of variation for three to five replicate injections of low and high concentration standards was consistently <3%. Potential rates of denitrification and N_2_O production were calculated based on the amounts of ^30^N_2_ and N_2_O, respectively, measured at T0 and T1 (1 h) after ^15^NO_3_ addition following the method described in Long et al. (2013). The samples with high N_2_ and N_2_O measured at T0 (three for N_2_ and six for N_2_O) were excluded in the rate calculation due to incomplete killing of microbial activities, which may be resulted from insufficient amount of KOH application.

### Molecular Analysis: Bacterial and Fungal Abundance

A subset of the soils stored at -80 °C (four pairs: 4 control and 4 tetracycline samples) was selected for microbial community analysis according to the measured activity rates to include the full range of N_2_ production inhibition (59 to 100% inhibition) by the tetracycline treatment. Genomic DNA was extracted from 0.5 g of soil using the PowerSoil DNA Isolation kit (MoBio). The DNA quality was assessed using a NanoDrop spectrometer (Thermo Scientific) and quantified with a Qubit^TM^ fluorometer (Invitrogen) and the dsDNA high-sensitivity kit. The abundance of bacteria and fungi was quantified by quantitative polymerase chain reaction (qPCR) of 16S rRNA and internal transcribed spacer (ITS) genes, respectively, using the QuantStudio 6 Flex (Thermo Scientific). Standards were prepared through a serial dilution of plasmids carrying the target genes and quantified using an Agilent 220 TapeStation System (Agilent Technologies). The primers EU341F (5′-CCT ACG GGA GGC AGC AG-3′) and 685R (5′-ATC TAC GGA TTT CAC TCC TAC A-3′) were used to generate 344 bp amplicons of bacterial 16S rRNA genes. The fungal ITS region was amplified using the primers ITS1F and ITS2 (Buee et al., 2009), generating 300–400 bp fragments. The 20 μL qPCR reactions for 16S rRNA and ITS quantification consisted of 10 μL of SYBR green Go-Taq qPCR Master Mix (Promega), 0.05 μL of CRX dye, 1 μL of each primer (10 μM), 2 ng of template DNA, and were adjusted to final volume with nuclease-free H_2_O. The qPCR conditions for 16S quantification were as follows: 10 min at 95°C, followed by 40 cycles of 15 s at 95°C, 30 s at 55°C, and 30 s at 72°C. Efficiency and *R*^2^ values for the 16S qPCR reaction were 63% and 0.99, respectively. The detection limit was 2400 gene copies per sample. For ITS quantification, the qPCR conditions were as follows: 10 min at 95°C, followed by 35 cycles of 15 s at 95°C, 30 s at 50°C, and 1 min at 72°C. Efficiency and *R*^2^ values for the ITS qPCR reaction were 55% and 0.97, respectively. The detection limit was 1170 gene copies per sample. All reactions were performed in 96 well plates with two negative controls, which contained no template DNA, to exclude any potential contamination. Reaction specificity was confirmed using gel electrophoresis in comparison with standards and monitored by analysis of dissociation curves. Gene copy number per PCR well was calculated from the standard curve according to the following equation:

$$copy\;number_{well}=10^{\frac{Ct-a}{b}}$$

Where Ct corresponds to the threshold cycle of the sample, and *a* and *b* correspond to the y-intercept and slope of the logarithmic standard curve, respectively. Copy numbers per well were then converted to copy number per gram of soil according to the following equation, assuming 100% DNA extraction efficiency:

$$\frac{gene\;copies}{g\;soil}={\left(\frac{copy\;number}{ng\;DNA}\right)}_{well}\times {\left(\frac{ng\;DNA}{\text{μ}L}\right)}_{sample}\times {\left(\frac{\text{μ}L\;DNA}{g\;soil}\right)}_{extracted}$$

### Molecular Analysis: Microbial Community Composition and Diversity

Next-generation sequencing of prokaryotic 16S rRNA and fungal ITS genes was used to examine the composition and diversity of prokaryotic and fungal communities in control and tetracycline samples. The communities were analyzed through barcode pyrosequencing using the Ion Torrent PGM sequencer. The variable V4 region of the 16S rRNA gene was amplified through PCR, using the forward primer 515F and a modified, barcoded reverse primer 806R (Caporaso et al., 2011). The ITS1 variable region of fungal ITS was amplified using a modified, barcoded forward primer ITS1F and the reverse primer ITS2 (White et al., 1990; Gardes and Bruns, 1993; Bellemain et al., 2010). The PCR mixture for 16S rRNA amplification contained 10 μL of Go-Taq mix, 1 μL of primers at 10 μM, 1 μL of template DNA (10–30 ng/μL), and nuclease-free H_2_O up to 25 μL. The PCR conditions for 16S rRNA amplification were as follows: 3 min at 95°C, followed by 25 cycles of 30 s at 95°C, 1 min at 55°C, and 1 min at 72°C, followed by 5 min at 72°C. The PCR mixture for ITS amplification contained 0.2 μL of Taq Polymerase (Invitrogen), 1 μL of primers at 10 M, 1 μL of template DNA (10–30 ng/μL), 2.5 μL of buffer (Invitrogen), 0.75 μL of dNTPs mix (1 mM), 1.0 μL of MgCl_2_ (50 mM), 0.25 μL of bovine serum albumin (BSA) at 10 mg/mL, and nuclease-free H_2_O up to 25 μL. The PCR conditions for ITS amplification were as follows: 4 min at 94°C, followed by 30 cycles of 30 s at 94°C, 1 min at 50°C, and 90 s at 72°C, followed by 10 min at 72°C. The fragment size of the 16S *rRNA* (354 bp) and ITS (363 bp) amplicons and negative control amplification were checked by 1% agarose gel electrophoresis. PCR products from each sample were pooled into a homogeneous mixture and a 2% agarose gel was run in duplicate to extract the amplicons, which were purified using an UltraClean GelSpin DNA Purification Kit (Promega). The concentration of purified amplicons was measured using a 2200 TapeStation instrument, following the manufacturer’s instruction. Pyrosequencing was conducted on the Ion Torrent PGM sequencer with barcode samples pooled on Ion 316 chips, following the Ion PGM Hi-Q Sequencing Kit protocol (Thermo Scientific).

### Bioinformatic Analysis: 16S rRNA and ITS sequences

Bioinformatic analysis of the 16S rRNA sequences was performed using the mothur program (Schloss et al., 2009). Primer sequences were trimmed, and all sequences shorter than 200 bp and with a quality score lower than 25 were removed. Acacia was used to de-noise the trimmed sequences (Bragg et al., 2012). The remaining sequences were then processed using mothur (Schloss et al., 2009). Unique sequences were found after alignment with the Silva SEED database (Quast et al., 2013). Badly aligned sequences were removed, unique sequences were pre-clustered, and chimeras were removed using UCHIME (Edgar et al., 2011). Sequences were classified using the SILVA v119 taxonomy, and unknown taxa were removed (Quast et al., 2013). Operational taxonomical units (OTUs) were clustered at 97% identity using the opticlust algorithm. Bacterial and archaeal OTUs were extracted for separate analysis of each community’s richness and diversity; samples were subsampled to the lowest number of sequences to normalize the diversity estimates. Chao and Ace indexes were calculated to estimate species richness, and Shannon was calculated to estimate α-diversity and community evenness. β-Diversity among samples was estimated using the Bray–Curtis dissimilarity calculator.

Fungal ITS sequence analysis was carried out using mothur and UPARSE (Schloss et al., 2009; Edgar, 2013). After initial processing of the FASTQ files using Acacia as described above, sequences were clustered into OTUs at 97% identity, using the UPARSE pipeline (Edgar, 2013). The samples were then subsampled to the lowest number of sequences and analyzed for species richness, diversity, and evenness, as described above for the prokaryotic community. Parallel to the OTU analysis, unique ITS sequences were classified using the UNITE v6_sh_97 ITS database in mothur (Abarenkov et al., 2010).

### Bioinformatic Analysis: Inference of *NosZ*-Carrying Bacteria Community Composition

Bacterial taxa carrying *nosZ* were identified based on a denitrification gene inference analysis on the rarefied bacterial 16S rRNA sequences (22,388 sequences per sample) using PAthway PRediction by phylogenetIC plAcement (PAPRICA) (Bowman and Ducklow, 2015; Arfken et al., 2017). A customized PAPRICA database was constructed with 8,513 complete and 785 draft bacterial genomes. The *nosZ* genes in the reference genomes were identified based on the KEGG database and used for gene prediction as described in Arfken et al. (2017). The estimated abundances of *nosZ*-carrying taxa were normalized to the number of 16S rRNA gene copies predicted for each taxon. Based on the taxonomy, the taxa carrying *nosZ*-I or *nosZ*-II were identified. Inferred abundances of *nosZ* clades per gram of soil were also calculated by multiplying relative abundances obtained through PAPRICA with the qPCR 16S copy numbers.

### Molecular Analysis: *NosZ*-I and *NosZ*-II Abundance

Quantitative PCR of *nosZ*-I and *nosZ*-II genes was also performed to measure the abundance of microorganisms responsible for the reduction of N_2_O to N_2_, using the QuantStudio 6 Flex (Thermo Scientific). Standards were prepared through a serial dilution of plasmids carrying the target genes and quantified using an Agilent 220 TapeStation System (Agilent Technologies). The primers used for *nosZ*-I genes were nosZ1F and nosZ1R and generated 300 bp amplicons (Henry et al., 2006). The *nosZ*-II genes were amplified using nosZIIF and nosZIIR primers that generated 690–720 bp amplicons (Jones et al., 2013). The 20 μL qPCR reactions for *nosZ*-I and *nosZ*-II quantification consisted of 10 μL of SYBR green Go-Taq qPCR Master Mix (Promega), 0.05 μL of CRX dye, 1 μL (*nosZ*-I) or 4 μL (*nosZ*-II) of each primer (10 μM), 2 ng of template DNA, and were adjusted to final volume with nuclease-free H_2_O. The thermal cycling conditions were the following: 10 min at 95°C, followed by 50 (*nosZ*-I) or 55 (*nosZ*-II) cycles of 15 s at 95°C, 45 s at 55°C (*nosZ*-I) or 30 s at 54°C (*nosZ*-II), 30 s at 72°C, and 35 s at 80°C for fluorescence detection. Amplification efficiencies were 46 and 40% for the *nosZ*-I and *nosZ*-II genes, respectively. The *R*^2^ value of the standard curves was 0.99 for both genes. The detection limit was 4,600 and 1,240 gene copies per sample for the *nosZ*-I and *nosZ*-II genes, respectively. All reactions were performed in 96 well plates with two negative controls, which contained no template DNA, to exclude any potential contamination. Reaction specificity was confirmed using gel electrophoresis in comparison with standards and monitored by analysis of dissociation curves during quantitative amplification. The gene copy numbers per gram of soil were calculated as described above for 16S rRNA gene and ITS.

### Statistical Analysis

Normality of all variables was assessed with Q–Q plots. Variables with large departures from normality were analyzed with non-parametric tests. A paired two-sample Mann–Whitney–Wilcoxon test (non-parametric) was used to identify significant differences between activity rates of control and tetracycline samples. Paired two-sample *t*-tests (parametric) were used to test for significant differences in 16S rRNA gene and ITS abundances, taxa relative abundances in prokaryotic and fungal communities, diversity estimators, and *nosZ*-I and *nosZ*-II gene abundances between control and tetracycline samples. Simple linear regressions were used to assess the relationship between *nosZ* abundances inferred by PAPRICA and determined by qPCR. Significant relationships for all tests were considered at α < 0.05. These statistical analyses were conducted in R (version 3.2.2. Copyright 2015 The R Foundation for Statistical Computing). Significant differences between control and tetracycline treatments in the abundance of *nosZ*-carrying taxa from PAPRICA analysis were tested by fitting the data into a generalized linear model (GLM) based on the negative binomial distribution using the DESeq function of the DESeq2 package in R (Love et al., 2014). A principal coordinate analysis (PCoA) was also performed to evaluate the β-diversity of bacterial, archaeal, and fungal communities using the phyloseq package in R (McMurdie and Holmes, 2013). Significant effects of the treatment in OTU dissimilarity among samples were tested by permutational multivariate ANOVA (PERMANOVA) using the adonis function of the vegan package in R (Oksanen et al., 2017).

## Results and Discussion

### Soil Properties

Average (standard deviation) bulk density, sand, silt, and clay contents for the 0–10 cm soil depth increment were 1.19 (0.04) g cm^-3^, and 730 (13), 230 (38) and 40 (30) g kg^-1^, respectively. At the time of soil collection, soil porosity was 55 (1.7) % and %WFPS was 38 (4). TOC and TN were 1.7 (0.2) and 0.2 (0.02)% dry weight (dw), respectively, and soil pH was 6.2 (0.1). Physicochemical factors potentially affecting microbial communities and denitrification activities were examined after the 1-week incubation period (**Table 1**). The only factor that was significantly different between the two groups was pH (paired *t*-test, *p* < 0.05). The tetracycline group (average pH = 6.9) had a significantly lower pH than the control group (average pH = 7.4). Despite this difference, the pH for both groups remained within the neutral region, where the 0.5 difference is not likely to significantly affect the denitrification end-products (Rochester, 2003; McMillan et al., 2016).

**Table 1:** Soil properties in samples incubated with and without tetracycline.

Treatment	Plot	pH	% organics	TOC (% dw)	TN (% dw)	${\text{NO}}_{3}^{-}$ (mg kg^-1^)	${\text{NO}}_{4}^{+}$ (mg Kg^-1^)
Control	1	7.32	6.31	2.34	0.21	21.27	21.10
	2	7.16	5.00	1.56	0.15	4.94	15.98
	3	7.69	4.44	1.65	0.18	6.40	15.02
	4	7.25	5.59	1.69	0.16	6.17	16.08
Tetracycline	1	7.05	5.65	2.42	0.25	150.83	15.02
	2	6.71	4.64	1.41	0.14	48.19	18.02
	3	7.04	4.64	1.49	0.14	15.60	11.46
	4	6.77	5.87	2.18	0.19	32.56	13.99
Paired t-test p-value		0.010	0.589	0.699	0.841	0.146	0.249

### Effects of Tetracycline on Denitrification: N_2_O and N_2_ Production

**FIGURE 1:**
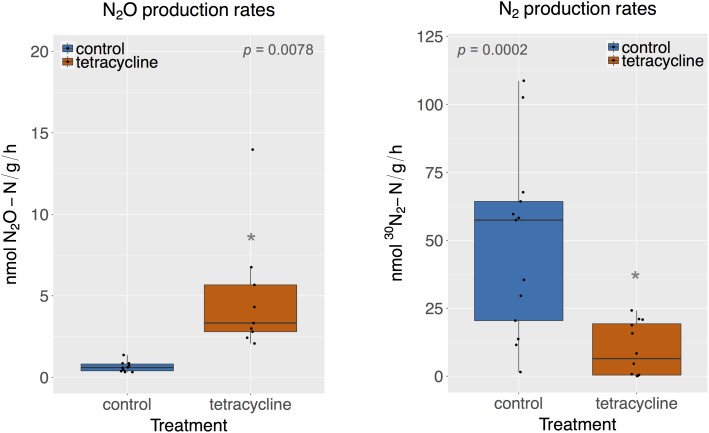
N_2_O and N_2_ production rates of control (blue) and tetracycline (red) samples. Significant difference between control and tetracycline treatment is marked with ^∗^ (paired Mann–Whitney-Wilcoxon test, *p* < 0.05). Each sample is represented by one point. The boxes represent the first and third quartiles, with median value bisecting each box. The whiskers extend to the largest/smallest value, excluding outliers (data beyond 1.5×inter-quartile range).

Rates of potential N_2_O and N_2_ production were significantly affected by tetracycline treatment, as shown in **Figure 1** (paired Mann–Whitney-Wilcoxon test, *p* < 0.05). N_2_O production in the tetracycline group ranged from 2.07 to 13.97 nmol N_2_O-N g^-1^ h^-1^, as compared to 0.32–1.37 nmol N_2_O-N g^-1^ h^-1^ in the control group. N_2_ production in the treated soil ranged from 0 to 24.2 nmol ^30^N_2_-N g^-1^ h^-1^, as compared to 1.52–108 nmol ^30^N_2_-N g^-1^ h^-1^ measured in the controls. Average N_2_O production was 12 times higher in the tetracycline group, while N_2_ production was inhibited by up to 84%. The N_2_O-N/N_2_-N ratio was 42 times higher in the tetracycline group than the controls. These results suggest that microbial reduction of N_2_O to N_2_ is strongly inhibited and N_2_O production is enhanced in soils exposed to tetracycline.

The stimulatory effect of antibiotics on N_2_O production has recently been observed in agricultural soils and estuarine sediments exposed to other antibiotics, such as sulfamethazine and narasin, for similar time periods and in lower concentrations than this study (DeVries et al., 2015; Hou et al., 2015; Yin et al., 2017). Observed increases in N_2_O production have been attributed to a shift in the end-products of denitrification from N_2_ to N_2_O driven by the stronger inhibition of N_2_O-reducing bacteria than N_2_O-producing bacteria (DeVries et al., 2015; Hou et al., 2015; Yin et al., 2017). However, different antibiotics, such as norfloxacin or a mixture of sulfadiazine, sulfamethoxazole, florfenicol, and chloramphenicol, were shown to reduce N_2_O production across various time periods and low exposure concentrations (Sun et al., 2017; Yin et al., 2017). The antibiotic’ effect on net N_2_O production seems to be dependent on the antibiotic type, dosage, and time of exposure, but no clear pattern has emerged in the literature. When considering tetracycline, we found only one study that measured how chronic exposure to tetracycline affected the production of different intermediates of denitrification in riverine sediments (Roose-Amsaleg et al., 2013). Roose-Amsaleg et al. (2013) reported no significant effect of tetracycline on ${\text{NO}}_{3}^{-}$ reduction, or ${\text{NO}}_{2}^{-}$ and N_2_O production at variable tetracycline concentrations up to 10 mg L^-1^. In our study, acute exposure to tetracycline in soil increased N_2_O and inhibited N_2_ production, indicating that the final denitrification step from N_2_O to N_2_ was susceptible to antibiotic exposure.

### Effects of Tetracycline on Bacterial and Fungal Abundance

**FIGURE 2:**
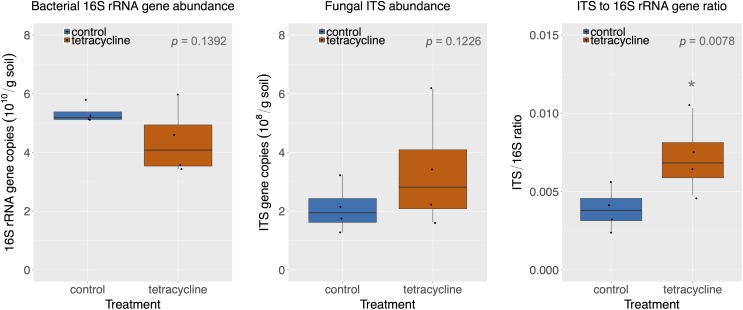
Comparison of bacterial 16S rRNA gene and fungal ITS abundance in control (blue) and tetracycline (red) samples. Significant difference between control and tetracycline treatment is marked with ^∗^ (paired *t*-test, *p* < 0.05). Each sample is represented by one point. The boxes represent the first and third quartiles, with median value bisecting each box. The whiskers extend to the largest/smallest value, excluding outliers (data beyond 1.5× inter-quartile range).

The abundance of bacterial 16S rRNA genes and fungal ITS is shown in **Figure 2**. The abundance levels observed in the control group ranged from 5.11 × 10^10^ to 5.79 × 10^10^ 16S rRNA gene copies per gram soil and from 1.27 × 10^8^ to 3.25 × 10^8^ ITS copies per gram soil. The tetracycline treatment had no significant effect (paired *t*-test, *p* > 0.05) on the abundance of either bacteria or fungi. However, the lowest levels of bacterial abundance and the highest levels of fungal abundance were observed in samples exposed to tetracycline, which resulted in a significant increase (paired *t*-test, *p* < 0.05) in the fungi:bacteria ratio in samples exposed to tetracycline compared to control (**Figure 2**).

The small subset of samples used for the molecular analysis (see section “Materials and Methods”) may have limited our capacity to detect smaller changes in bacterial or fungal absolute abundances. However, the subset used was enough to detect significant changes in the fungi:bacteria ratio, as hypothesized. Previous studies showed significant increases in the fungi:bacteria ratio in soils exposed to antibiotics, including tetracyclines (Thiele-Bruhn and Beck, 2005; Hammesfahr et al., 2008; Demoling et al., 2009; Ding and He, 2010; Gutiérrez et al., 2010). This is expected since tetracycline inhibits bacterial growth and may leave nutrients and habitat for fungal growth. The increase in the fungi:bacteria ratio suggests that the higher N_2_O production observed could be associated with fungal denitrification (Shoun et al., 2012; Maeda et al., 2015; Mothapo et al., 2015). Various studies have shown that fungal denitrification is a major contributor to N_2_O production in different soils (Laughlin and Stevens, 2002; Long et al., 2013; Wei et al., 2014; Chen et al., 2015; Huang et al., 2017).

### Effects of Tetracycline on Prokaryotic Community Composition

**FIGURE 3:**
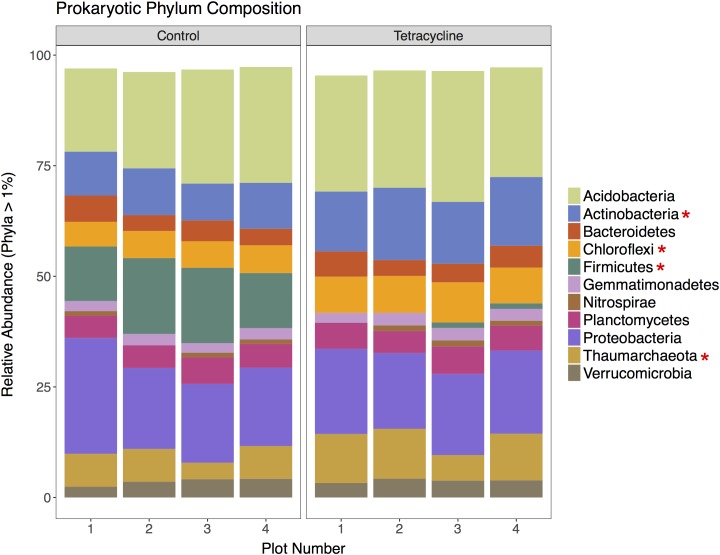
Prokaryotic community composition at the phylum level for control and tetracycline samples. Only phyla with relative abundance higher than 1% are shown. Phyla with significant difference between control and tetracycline samples are marked with ^∗^ in the legend (paired *t*-test, *p* < 0.05).

A total of 201,980 16S rRNA gene sequences were obtained for taxonomic analysis following screening and filtering of the prokaryotic sequences. Bacteria was clearly the most abundant domain with relative abundances ranging from 89 to 96%, while archaeal sequences represented less than 12% of the communities. The tetracycline treatment shifted the relative abundance away from Bacteria (paired *t*-test, *p* = 0.00426) and toward Archaea (paired *t*-test, *p* = 0.00440) by 4%. This was expected since tetracycline is an antibacterial compound that would mostly inhibit bacterial growth. The relative abundances of the most represented prokaryotic phyla (16S rRNA > 1% of total reads) in control and tetracycline groups are shown in **Figure 3**. Eleven abundant phyla were identified. All samples except one were dominated by Acidobacteria, representing 19–30% of the sequences, followed by Proteobacteria (17–26%), Firmicutes (0.5–17%), Actinobacteria (8.3–16%), and Thaumarchaeota (3.7–11%). The prokaryotic phyla that were significantly different between control and tetracycline groups were Actinobacteria (paired *t*-test, *p* = 0.00203), Chloroflexi (paired *t*-test, *p* = 0. 00287), Firmicutes (paired *t*-test, *p* = 0.00210), and Thaumarchaeota (paired *t*-test, *p* = 0.00430). The most drastic effect was the decline in the relative abundance of Firmicutes, from an average of 14% in controls to 0.9% in the tetracycline samples. The Firmicutes classes most affected by the tetracycline treatment were Bacilli and Clostridia (**Supplementary Figure S1**). These results show that specific bacterial taxa are more susceptible to tetracycline exposure despite its broad-spectrum antibacterial properties.

Firmicutes is likely to be less resistant to tetracycline according to previous studies, which report a decrease in the ratio of Gram-positive:Gram-negative bacteria when exposed to tetracycline and other antimicrobials (Hund-Rinke et al., 2004; Ding and He, 2010; Gutiérrez et al., 2010). Indeed, the vast majority of tetracycline-resistant bacteria are Gram-negative (Schnabel and Jones, 1999). The observed decrease in the relative abundance of Firmicutes may have important consequences for N_2_O reduction since bacteria harboring *nosZ* genes without possessing any *nir* or *nor* gene are mainly found amongst the Bacteroidetes and Firmicutes phyla (Graf et al., 2014).

### Effects of Tetracycline on Fungal Community Composition

**FIGURE 4:**
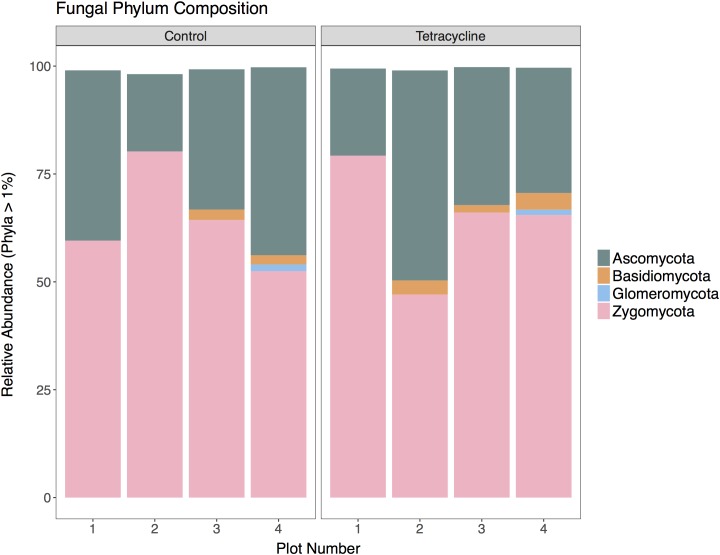
Fungal community composition at the phylum level for control and tetracycline samples. Only phyla with relative abundance higher than 1% are shown. Taxa with significant difference between control and tetracycline samples are marked with ^∗^ in the legend (paired *t*-test, *p* < 0.05).

A total of 369,805 (ITS) sequences were obtained for taxonomic analysis following screening and filtering of the fungal sequences. The relative abundances of the most represented fungal phyla (ITS > 1% of total reads) in control and tetracycline groups are shown in **Figure 4**. Five abundant phyla were identified. All samples were dominated by Zygomycota, representing 47–80% of the sequences, followed by Ascomycota (18–49%). The tetracycline treatment did not have significant effects (paired *t*-test, *p* > 0.05) on the relative abundance of any fungal phyla, including the N_2_O producing Ascomycota, Basidiomycota, and Zygomycota (Maeda et al., 2015; Mothapo et al., 2015). Most fungal denitrifiers belong to the Sordariomycetes, Eurotiomycetes, and Saccharomycetes classes (Mothapo et al., 2015). No significant differences between treatments were detected in the relative abundance of any of these classes (**Supplementary Figure S2**). Although the present study shows a tetracycline impact on N_2_O production and the fungi:bacteria abundance ratio, no individual impacts appear to occur in fungal denitrifier taxa.

### Effects of Tetracycline in the Community Richness and Diversity

**FIGURE 5:**
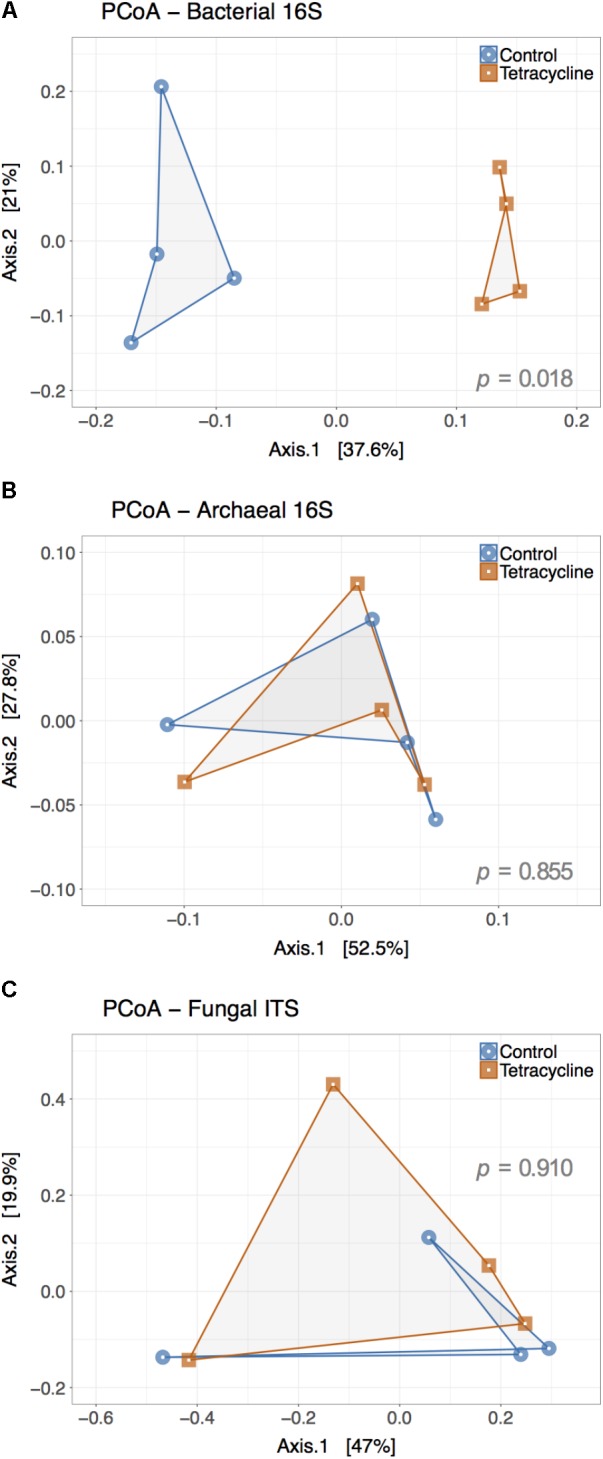
Principal Coordinate Analysis (PCoA) plot representing the β-diversity of the bacterial **(A)**, archaeal **(B)**, and fungal **(C)** communities from control (blue) and tetracycline (red) samples. Both bacterial and archaeal communities were examined based on 16S rRNA gene sequences while ITS sequences were used for fungal communities. Sample dissimilarity and distance analysis was calculated using the Bray–Curtis dissimilarity index. Significant effects (*p* < 0.05) of the treatment in OTU dissimilarity were tested by multivariate permutational ANOVA (PERMANOVA) and the *p*-values are shown in each plot.

Principle coordinate analysis represents β-diversity of microbial communities based on the calculated dissimilarities among samples (**Figure 5**). The first two principal coordinates represented 58.6% and 80.3% of the variation in bacterial and archaeal communities (**Figures 5A,B**), respectively. The archaeal β-diversity was not significantly affected by the tetracycline treatment (PERMANOVA, *p* > 0.05), while the bacterial communities had a distinct clustering of control samples separated from the tetracycline samples. This result shows a significant difference (PERMANOVA, *p* < 0.05) between the compositions of bacterial communities in control and tetracycline groups. Previous studies also reported significant effects of different antibiotics (sulfadiazine, tylosin, amoxicillin, and ciprofloxacin) on bacterial community structure in soils and marine sediments (Westergaard et al., 2001; Zielezny et al., 2006; Binh et al., 2007; Hammesfahr et al., 2008; Näslund et al., 2008). When considering tetracyclines, Zielezny et al. (2006) did not find significant effects in the bacterial community structure after exposure to 1–50 mg kg^-1^ of chlortetracycline for 48 h. This result contrasts with our study, where the effects of tetracycline on bacterial community structure were significant, probably due to the higher dosage and longer duration of exposure tested (1 week). Since tetracyclines are protein synthesis inhibitors with bacteriostatic action (i.e., limits bacterial growth) rather than bactericidal it is possible that an incubation period longer than 48 h is necessary to detect changes in community structure.

Contrasting to the bacterial communities, no clear patterns were observed in fungal β-diversity (**Figure 5C**). The first two principal coordinates represented 66.9% of the variation in fungal communities and no significant effect of treatment was observed (PERMANOVA, *p* > 0.05). This result is consistent with the observed lack of significant changes in the relative abundance of the most abundant fungal phyla and classes.

The effects of tetracycline on species richness and microbial α-diversity are displayed in **Table 2**. Interestingly, bacterial α-diversity, estimated through calculation of the Shannon Index (H′), was significantly higher (paired *t*-test, *p* < 0.05) in the tetracycline group (average *H*′ = 6.36) when compared to controls (average *H*′ = 5.98). An increase in bacterial diversity after antibiotic exposure was not expected since previous studies have shown that antibiotic exposure decreases bacterial diversity in the soil ecosystem (Westergaard et al., 2001; Zielezny et al., 2006; Cycoñ et al., 2016; Ding and He, 2010). However, temporary increases (after 4 days of exposure) in bacterial diversity of agricultural soils exposed to antibiotics have also been reported (Hammesfahr et al., 2008). The increase in bacterial diversity was probably caused by the inhibition of the most dominant taxa and a slight increase in the numbers of rare taxa. This explanation is supported by the significant increase in community evenness (paired *t*-test, *p* < 0.05), estimated through calculation of the Shannon index-based evenness (E_H_), and the non-significant difference observed in both indexes of species richness (Chao and Ace; paired *t*-test, *p* > 0.05). We found that higher community diversity in the tetracycline group was associated with higher N_2_O emission, while previous studies showed that these two variables are negatively correlated (Wagg et al., 2014; Samad et al., 2016). These studies, however, tested a broader range of bacterial diversity and statistically tested the direct relationships between microbial diversity and N_2_O emissions. In our study, the observed higher bacterial diversity in the tetracycline group with higher N_2_O production could be sporadic and the relationship between microbial diversity and N_2_O emissions is outside of the scope of this work.

**Table 2:** Species richness and α-diversity of microbial communities from control and tetracycline samples.

Microbial Group	Treatment	Plot	Sequences	OTUs	Coverage	Richness		Diversity	Evenness
						Chao	Ace	Shannon	Shannon
**Bacteria (16S)**	Control	1	22388	2986	0.931	5820.3	7617.0	6.05	0.76
		2	22388	2734	0.937	5159.3	7209.6	5.82	0.74
		3	22388	2636	0.939	5047.7	7195.1	5.98	0.76
		4	22388	2767	0.937	5236.3	7032.6	6.09	0.77
	Tetracycline	1	22388	3052	0.931	5749.6	7511.0	6.44	0.80
		2	22388	2874	0.933	5462.4	7641.0	6.27	0.79
		3	22388	3005	0.928	6126.8	8868.8	6.35	0.79
		4	22388	2944	0.933	5446.9	7486.5	6.39	0.80
Paired t-test p-value						0.220	0.202	0.001	0.003
**Archaea (16S)**	Control	1	946	16	0.994	23.5	29.3	1.88	0.68
		2	946	19	0.989	41.5	74.0	2.02	0.69
		3	946	19	0.990	31.0	43.8	1.95	0.66
		4	946	17	0.993	38.0	73.9	2.05	0.72
	Tetracycline	1	946	12	0.998	13.0	13.9	1.85	0.74
		2	946	15	0.996	16.5	19.7	2.00	0.74
		3	946	14	0.995	19.0	29.0	1.96	0.74
		4	946	15	0.996	17.0	20.1	2.04	0.75
Paired t-test p-value						0.016	0.054	0.200	0.016
**Fungi (ITS)**	Control	1	25863	261	0.999	276.3	271.5	3.28	0.59
		2	25863	224	0.999	232.0	231.3	2.42	0.45
		3	25863	174	0.999	180.5	181.1	3.18	0.62
		4	25863	238	0.999	249.8	247.7	3.56	0.65
	Tetracycline	1	25863	220	0.999	239.9	235.5	2.64	0.49
		2	25863	192	0.999	200.3	199.6	3.65	0.69
		3	25863	196	0.999	207.1	212.7	2.79	0.53
		4	25863	269	0.999	277.1	279.5	3.44	0.62
Paired t-test p-value						0.853	0.958	0.960	0.942

Similarly to the β-diversity patterns, no significant changes (paired *t*-test, *p* > 0.05) were detected in fungal species richness, α-diversity, or evenness with tetracycline exposure (**Table 2**). As expected, tetracycline had no impact on the composition of the fungal communities at any taxonomical level.

### Effects of Tetracycline on *NosZ*-Carrying Community Composition

By employing metabolic inference using PAPRICA (Bowman and Ducklow, 2015), we were able to identify the 16S rRNA sequences belonging to the bacterial taxa carrying either *nosZ*-I or *nosZ*-II genes. Within the *nosZ*-I community, Gammaproteobacteria was the most abundant class, followed by Alphaproteobacteria (Proteobacteria) (**Supplementary Figure S3**). A few sequences belonging to the Caldilineae class (Chloroflexi) and Betaproteobacteria (Proteobacteria) were also observed in this study. The *nosZ*-II communities were more taxonomically diverse at the phylum and class levels (**Supplementary Figure S4**). Sixteen classes of *nosZ*-II-carrying taxa were identified and virtually all samples were dominated by Betaproteobacteria (Bacteroidetes), Deltaproteobacteria (Proteobacteria), and Opitutae (Verrucomicrobia).

**FIGURE 6:**
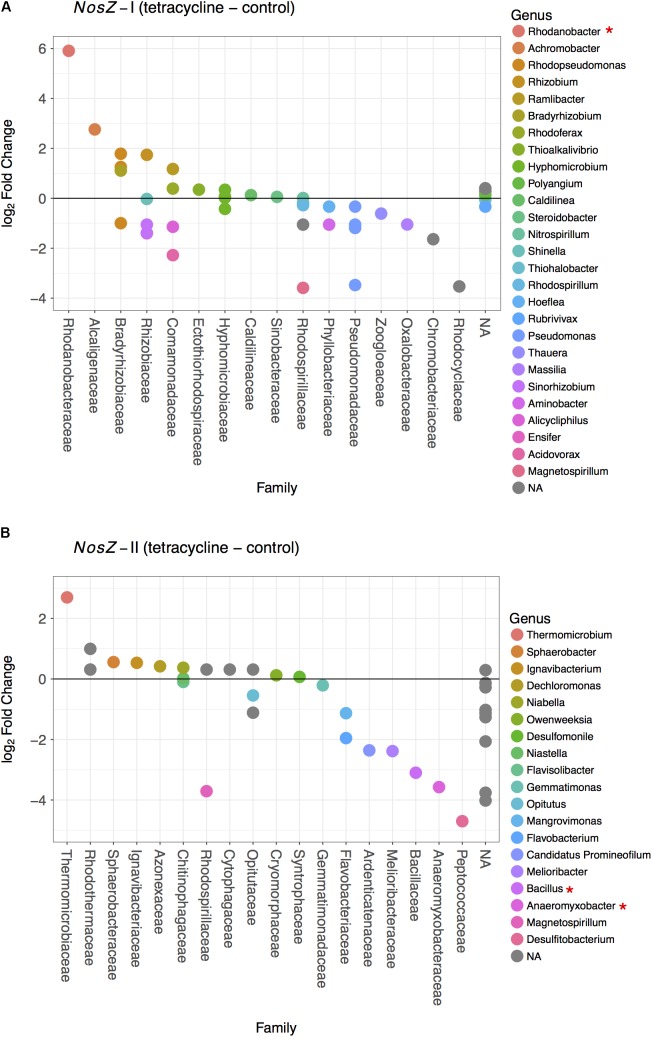
Difference in the *nosZ*-carrying microbial community composition between tetracycline and control samples for *nosZ*-I **(A)** and *nosZ*-II **(B)** bacteria. Each dot denotes a taxon that is either higher (log_2_ fold change > 0) or lower (log_2_ fold change < 0) in the tetracycline treated samples when compared to the control samples. Taxa are organized by family in decreasing order and colored by genus. Normalized abundances were obtained by metabolic inference of 16S rRNA gene sequences using PAPRICA and tested for significant effects (*p* < 0.05) of treatment by fitting the data into a generalized linear model (GLM) based on the negative binomial distribution. Taxa with significant difference between tetracycline and control samples are marked with ^∗^ in the legend.

The effects of tetracycline on *nosZ*-carrying taxa were evaluated based on the changes in families and genera of bacteria carrying *nosZ*-I or *nosZ*-II genes (**Figure 6**). The tetracycline group had a significantly higher abundance of the *nosZ*-I carrying *Rhodanobacter* spp. (Gammaproteobacteria) than the control group (negative binomial GLM, *p* = 0.0038) (**Figure 6A**). On the other hand, the tetracycline treatment significantly decreased the abundance of two *nosZ*-II carrying taxa, *Bacillus* spp. (Firmicutes; negative binomial GLM, *p* = 0.0008) and *Anaeromyxobacter* spp. (Deltaproteobacteria; negative binomial GLM, *p* = 0.0375) (**Figure 6B**). Abundances of both taxa were approximately eight times lower in the tetracycline group.

The observed decrease in *nosZ*-II-carrying *Bacillus* spp. is consistent with the decrease in relative abundance of Firmicutes and provides evidence that some of the affected Firmicutes bacteria were potential *nosZ*-II carriers. The microbes harboring *nosZ* genes without possessing any *nir* or *nor* gene are frequently found amongst the Firmicutes phylum (Graf et al., 2014). This underscores the importance of some *nosZ*-carrying Firmicutes as N_2_O consumers, rather than N_2_O producers, in the soil ecosystem. The other genus that had lower abundances in the tetracycline group, *Anaeromyxobacter* spp. is also known as a non-denitrifying N_2_O reducer, i.e., these bacteria do not possess all other denitrification genes besides *nosZ* (Sanford et al., 2012; Hallin et al., 2017). This finding suggests that non-denitrifying N_2_O reducers are more susceptible to antibiotic disturbance than denitrifying N_2_O reducers. Both *nosZ*-II taxa significantly affected by the tetracycline exposure are likely important N_2_O consumers rather than N_2_O producers. The observed decreases in abundances of *Bacillus* (Firmicutes) and *Anaeromyxobacter* (Deltaproteobacteria) may thus explain the observed increase in N_2_O/N_2_ ratio in the same treatment. These decreases also indicate that, despite tetracycline being a broad-spectrum antibiotic, there are functional bacterial taxa more susceptible than others.

### Effects of Tetracycline on Bacterial *nosZ* Abundance

**FIGURE 7:**
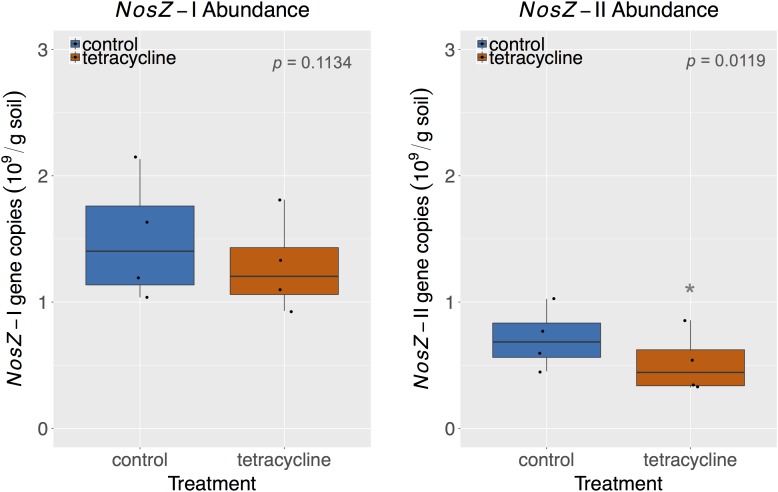
*NosZ*-I and *nosZ*-II abundance measured by quantitative PCR (qPCR) in control (blue) and tetracycline (red) samples. Significant difference between control and tetracycline treatment is marked with ^∗^ (paired *t*-test, *p* < 0.05). Each sample is represented by one point. The boxes represent the first and third quartiles, with median value bisecting each box. The whiskers extend to the largest/smallest value, excluding outliers (data beyond 1.5× inter-quartile range).

The abundances of *nosZ*-I and *nosZ*-II genes measured by qPCR are shown in **Figure 7**. Abundances of *nosZ*-I and *nosZ*-II genes in the control group ranged from 1.04 to 2.13 × 10^9^ copies per gram soil and from 0.45 to 1.03 × 10^9^ copies per gram soil, respectively. The *nosZ*-I abundance was not significantly affected by tetracycline (paired *t*-test, *p* > 0.05), while the *nosZ*-II abundance was significantly lower (paired *t*-test, *p* < 0.05) in the tetracycline group (**Figure 7**).

Together, *nosZ*-I and *nosZ*-II comprised 2.9–5.5 % of the total number of 16S rRNA gene copies, which are in accordance with reports in the literature for *nosZ* relative abundances (Jones et al., 2013, 2014; Domeignoz-Horta et al., 2015). Taking the effect of tetracycline into account, the *nosZ*-II community appears to be more sensitive to the antibiotic than the *nosZ*-I community. This result supports the observed taxonomical changes described above for the *nosZ*-carrying taxa. The *nosZ*-II community was previously reported to be more sensitive than *nosZ*-I to other environmental factors, such as pH, calcium concentration, soil moisture, total N, and crop rotation systems (Jones et al., 2014; Domeignoz-Horta et al., 2015; Samad et al., 2016). The significant decrease of *nosZ*-II abundance reported here for samples exposed to tetracycline corresponds to the observed increase in N_2_O/N_2_ ratio in the same treatment, which underscores the importance of this clade as N_2_O consumers.

**FIGURE 8:**
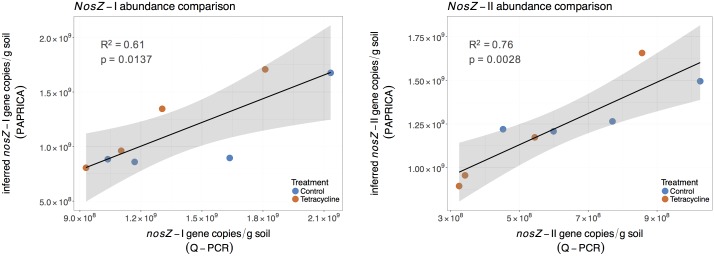
Linear regressions comparing inferred and quantified *nosZ* gene abundances for control (blue) and tetracycline (red) samples. The inferred *nosZ* abundances were calculated by multiplying the relative abundances obtained through metabolic inference (PAPRICA) with the 16S rRNA gene abundances. The shaded area represents the 95% confidence interval of the linear regression predictions.

Abundance of *nosZ*-I and *nosZ*-II genes measured by qPCR were compared with the inferred abundance of both genes by PAPRICA (**Figure 8**). A significant, positive linear correlation was observed between the two estimates of the *nosZ* clades. The inferred abundances of *nosZ*-I were on average 17% lower than those determined by qPCR, while inferred *nosZ*-II abundances were on average two times higher than qPCR results.

A similar significant relationship between inferred and qPCR abundances was previously reported for the *nosZ*-I gene in the oyster microbiome (Arfken et al., 2017). These findings suggest that 16S rRNA gene-based metabolic inference is a promising approach to evaluate functional gene abundances and community composition in environmental samples. Despite the small sampling size of our study and the intrinsic limitations of a reference database, this approach could be of great relevance to the microbial ecology field as 16S rRNA gene high throughput sequencing becomes increasingly available.

## Conclusion

We present several lines of evidence that unveil the microbial community changes associated with the antibiotic impacts on soil denitrification. The increase in N_2_O/N_2_ ratio from denitrification is paralleled by a greater fungi:bacteria ratio and lower abundance of *nosZ*-II carrying bacteria. Non-denitrifying N_2_O reducers belonging to the Firmicutes and Deltaproteobacteria phyla appear to be particularly susceptible to an acute exposure to tetracycline and may be crucial for soil N_2_O sink capacity. Future research aiming to predict N_2_O emissions based on microbial community structure under antibiotic-disturbed environments would potentially benefit from targeted approaches to these taxa. Overall, the findings of this study emphasize the importance of microbial community dynamics to fundamental ecosystem processes that can have major impacts on the emissions of a potent greenhouse gas such as N_2_O.

## Author Contributions

BS and RP conceived and designed the experiments. MS, BS, and TS performed the experiments and acquired the data. MS analyzed the data. MS, BS, and RP wrote the paper. All authors approved the final submitted manuscript.

## Conflict of Interest Statement

The authors declare that the research was conducted in the absence of any commercial or financial relationships that could be construed as a potential conflict of interest.

## References

[B1] Abarenkov K. Henrik Nilsson R. Larsson K.-H. Alexander I. J. Eberhardt U. Erland S. (2010). The UNITE database for molecular identification of fungi – recent updates and future perspectives. *New Phytol.* 186 281–285. 10.1111/j.1469-8137.2009.03160.x 20409185

[B2] Ahmad M. Vithanage M. Kim K. Cho J. S. Lee Y. H. Joo Y. K. (2014). Inhibitory effect of veterinary antibiotics on denitrification in groundwater: a microcosm approach. *Sci. World J.* 2014 879831. 10.1155/2014/879831 24757442 PMC3976856

[B3] Arfken A. Song B. Bowman J. S. Piehler M. (2017). Denitrification potential of the eastern oyster microbiome using a 16S rRNA gene based metabolic inference approach. *PLoS One* 12:e0185071. 10.1371/journal.pone.0185071 28934286 PMC5608302

[B4] Bellemain E. Carlsen T. Brochmann C. Coissac E. Taberlet P. Kauserud H. (2010). ITS as an environmental DNA barcode for fungi: an in silico approach reveals potential PCR biases. *BMC Microbiol.* 10:189. 10.1186/1471-2180-10-189 20618939 PMC2909996

[B5] Binh C. T. T. Heuer H. Gomes N. C. M. Kotzerke A. Fulle M. Wilke B.-M. (2007). Short-term effects of amoxicillin on bacterial communities in manured soil. *FEMS Microbiol. Ecol.* 62 290L–302L. 10.1111/j.1574-6941.2007.00393.x 17991020

[B6] Bowman J. S. Ducklow H. W. (2015). Microbial communities can be described by metabolic structure: a general framework and application to a seasonally variable, depth-stratified microbial community from the coastal West Antarctic Peninsula. *PLoS One* 10:e0135868. 10.1371/journal.pone.0135868 26285202 PMC4540456

[B7] Boxall A. B. A. (2004). The environmental side effects of medication. *EMBO Rep.* 5 1110–1116. 10.1038/sj.embor.7400307 15577922 PMC1299201

[B8] Bragg L. Stone G. Imelfort M. Hugenholtz P. Tyson G. W. (2012). Fast, accurate error-correction of amplicon pyrosequences using Acacia. *Nat. Methods* 9 425–426. 10.1038/nmeth.1990 22543370

[B9] Buee M. Reich M. Murat C. Morin E. Nilsson R. H. Uroz S. (2009). 454 Pyrosequencing analyses of forest soils reveal an unexpectedly high fungal diversity. *New Phytol.* 184 449–456. 10.1111/j.1469-8137.2009.03003.x 19703112

[B10] Caporaso J. G. Lauber C. L. Walters W. A. Berg-Lyons D. Lozupone C. A. Turnbaugh P. J. (2011). Global patterns of 16S rRNA diversity at a depth of millions of sequences per sample. *Proc. Natl. Acad. Sci. U.S.A.* 108(Suppl.), 4516–4522. 10.1073/pnas.1000080107 20534432 PMC3063599

[B11] Chen H. Mothapo N. V. Shi W. (2015). Fungal and bacterial N2O production regulated by soil amendments of simple and complex substrates. *Soil Biol. Biochem.* 84 116–126. 10.1016/j.soilbio.2015.02.018

[B12] Conkle J. L. White J. R. (2012). An initial screening of antibiotic effects on microbial respiration in wetland soils. *J. Environ. Sci. Health A Tox. Hazard. Subst. Environ. Eng.* 47 1381–1390. 10.1080/10934529.2012.672315 22571526

[B13] Costanzo S. D. Murby J. Bates J. (2005). Ecosystem response to antibiotics entering the aquatic environment. *Mar. Pollut. Bull.* 51 218–223. 10.1016/j.marpolbul.2004.10.038 15757723

[B14] Cycoñ M. Borymski S. Orlewska K. Wasik T. J. Piotrowska-Seget Z. (2016). An analysis of the effects of vancomycin and/or vancomycin-resistant *Citrobacter freundii* exposure on the microbial community structure in soil. *Front. Microbiol.* 7:1015 10.3389/fmicb.2016.01015 PMC4923127 27446053

[B15] Daghrir R. Drogui P. (2013). Tetracycline antibiotics in the environment: a review. *Environ. Chem. Lett.* 11 209–227. 10.1007/s10311-013-0404-8

[B16] Demoling L. A. Bååth E. Greve G. Wouterse M. Schmitt H. (2009). Effects of sulfamethoxazole on soil microbial communities after adding substrate. *Soil Biol. Biochem.* 41 840–848. 10.1016/j.soilbio.2009.02.001

[B17] DeVries S. L. Loving M. Li X. Zhang P. (2015). The effect of ultralow-dose antibiotics exposure on soil nitrate and N2O flux. *Sci. Rep.* 5:16818. 10.1038/srep16818 26606964 PMC4660347

[B18] Ding C. He J. (2010). Effect of antibiotics in the environment on microbial populations. *Appl. Microbiol. Biotechnol.* 87 925–941. 10.1007/s00253-010-2649-5 20508933

[B19] Domeignoz-Horta L. A. Spor A. Bru D. Breuil M.-C. Bizouard F. Léonard J. (2015). The diversity of the N(2)O reducers matters for the N(2)O:N(2) denitrification end-product ratio across an annual and a perennial cropping system. *Front. Microbiol.* 6:971 10.3389/fmicb.2015.00971 PMC4585238 26441904

[B20] Edgar R. C. (2013). UPARSE: highly accurate OTU sequences from microbial amplicon reads. *Nat. Methods* 10 996–998. 10.1038/nmeth.2604 23955772

[B21] Edgar R. C. Haas B. J. Clemente J. C. Quince C. Knight R. (2011). UCHIME improves sensitivity and speed of chimera detection. *Bioinformatics* 27 2194–2200. 10.1093/bioinformatics/btr381 21700674 PMC3150044

[B22] Gardes M. Bruns T. D. (1993). ITS primers with enhanced specificity for basidiomycetes - application to the identification of mycorrhizae and rusts. *Mol. Ecol.* 2 113–118. 10.1111/j.1365-294X.1993.tb00005.x 8180733

[B23] Graf D. R. H. Jones C. M. Hallin S. (2014). Intergenomic comparisons highlight modularity of the denitrification pathway and underpin the importance of community structure for N2O emissions. *PLoS One* 9:e114118. 10.1371/journal.pone.0114118 25436772 PMC4250227

[B24] Gutiérrez I. R. Watanabe N. Harter T. Glaser B. Radke M. (2010). Effect of sulfonamide antibiotics on microbial diversity and activity in a californian mollic haploxeralf. *J. Soils Sediments* 10 537–544. 10.1007/s11368-009-0168-8

[B25] Hallin S. Philippot L. Löffler F. E. Sanford R. A. Jones C. M. (2017). Genomics and ecology of novel N2O-reducing microorganisms. *Trends Microbiol.* 26 43–55. 10.1016/j.tim.2017.07.003 28803698

[B26] Hammesfahr U. Heuer H. Manzke B. Smalla K. Thiele-Bruhn S. (2008). Impact of the antibiotic sulfadiazine and pig manure on the microbial community structure in agricultural soils. *Soil Biol. Biochem.* 40 1583–1591. 10.1016/j.soilbio.2008.01.010

[B27] Henry S. Bru D. Stres B. Hallet S. Philippot L. (2006). Quantitative detection of the *nosZ* gene, encoding nitrous oxide reductase, and comparison of the abundances of 16S rRNA, *narG, nirK*, and *nosZ* Genes in Soils. *Appl. Environ. Microbiol.* 72 5181–5189. 10.1128/AEM.00231-06 16885263 PMC1538733

[B28] Hou L. Yin G. Liu M. Zhou J. Zheng Y. Gao J. (2015). Effects of sulfamethazine on denitrification and the associated N2O release in estuarine and coastal sediments. *Environ. Sci. Technol.* 49 326–333. 10.1021/es504433r 25525860

[B29] Huang Y. Xiao X. Long X. (2017). Fungal denitrification contributes significantly to N2O production in a highly acidic tea soil. *J. Soils Sediments* 17 1599–1606. 10.1007/s11368-017-1655-y

[B30] Hund-Rinke K. Simon M. Lukow T. (2004). Effects of tetracycline on the soil microflora: function, diversity, resistance. *J. Soils Sediments* 4 11–16. 10.1007/BF02990823

[B31] Jones C. M. Graf D. R. H. Bru D. Philippot L. Hallin S. (2013). The unaccounted yet abundant nitrous oxide-reducing microbial community: a potential nitrous oxide sink. *ISME J.* 7 417–426. 10.1038/ismej.2012.125 23151640 PMC3554408

[B32] Jones C. M. Spor A. Brennan F. P. Breuil M.-C. Bru D. Lemanceau P. (2014). Recently identified microbial guild mediates soil N2O sink capacity. *Nat. Clim. Chang.* 4 801–805. 10.1038/nclimate2301

[B33] Kleineidam K. Sharma S. Kotzerke A. Heuer H. Thiele-Bruhn S. Smalla K. (2010). Effect of sulfadiazine on abundance and diversity of denitrifying bacteria by determining nirK and nirS genes in two arable soils. *Microb. Ecol.* 60 703–707. 10.1007/s00248-010-9691-9 20532498

[B34] Kotzerke A. Sharma S. Schauss K. Heuer H. Thiele-Bruhn S. Smalla K. (2008). Alterations in soil microbial activity and N-transformation processes due to sulfadiazine loads in pig-manure. *Environ. Pollut.* 153 315–322. 10.1016/j.envpol.2007.08.020 17905496

[B35] Laughlin R. J. Stevens R. J. (2002). Evidence for fungal dominance of denitrification and codenitrification in a grassland soil. *Soil Sci. Soc. Am. J.* 66:1540 10.2136/sssaj2002.1540

[B36] Long A. Heitman J. Tobias C. Philips R. Song B. (2013). Co-occurring anammox, denitrification, and codenitrification in agricultural soils. *Appl. Environ. Microbiol.* 79 168–176. 10.1128/AEM.02520-12 23087029 PMC3536082

[B37] Love M. I. Huber W. Anders S. (2014). Moderated estimation of fold change and dispersion for RNA-seq data with DESeq2. *Genome Biol.* 15:550. 10.1186/s13059-014-0550-8 25516281 PMC4302049

[B38] Ma W. K. Farrell R. E. Siciliano S. D. (2011). Nitrous oxide emissions from ephemeral wetland soils are correlated with microbial community composition. *Front. Microbiol.* 2:110. 10.3389/fmicb.2011.00110 21712943 PMC3114181

[B39] Maeda K. Spor A. Edel-Hermann V. Heraud C. Breuil M.-C. Bizouard F. (2015). N2O production, a widespread trait in fungi. *Sci. Rep.* 5:9697. 10.1038/srep09697 25894103 PMC4403702

[B40] Massé D. I. Cata Saady N. M. Gilbert Y. (2014). Potential of biological processes to eliminate antibiotics in livestock manure: an overview. *Animals* 4 146–163. 10.3390/ani4020146 26480034 PMC4494381

[B41] McMillan A. M. S. Pal P. Phillips R. L. Palmada T. Berben P. H. Jha N. (2016). Can pH amendments in grazed pastures help reduce N2O emissions from denitrification? – The effects of liming and urine addition on the completion of denitrification in fluvial and volcanic soils. *Soil Biol. Biochem.* 93 90–104. 10.1016/j.soilbio.2015.10.013

[B42] McMurdie P. J. Holmes S. (2013). Phyloseq: an r package for reproducible interactive analysis and graphics of microbiome census data. *PLoS One* 8:e61217. 10.1371/journal.pone.0061217 23630581 PMC3632530

[B43] Mothapo N. Chen H. Cubeta M. A. Grossman J. M. Fuller F. Shi W. (2015). Phylogenetic, taxonomic and functional diversity of fungal denitrifiers and associated N2O production efficacy. *Soil Biol. Biochem.* 83 160–175. 10.1016/j.soilbio.2015.02.001

[B44] Näslund J. Hedman J. E. Agestrand C. (2008). Effects of the antibiotic ciprofloxacin on the bacterial community structure and degradation of pyrene in marine sediment. *Aquat. Toxicol.* 90 223–227. 10.1016/j.aquatox.2008.09.002 18930559

[B45] Neubauer S. C. Megonigal J. P. (2015). Moving beyond global warming potentials to quantify the climatic role of ecosystems. *Ecosystems* 18 1000–1013. 10.1007/s10021-015-9879-4

[B46] Oksanen J. Blanchet F. G. Friendly M. Kindt R. Legendre P. McGlinn D. (2017). *vegan: Community Ecology Package.* Available at: https://cran.r-project.org/package=vegan.

[B47] Phillips R. L. Eken M. R. West M. S. (2015). Soil organic carbon beneath croplands and re-established grasslands in the north dakota prairie pothole region. *Environ. Manage.* 55 1191–1199. 10.1007/s00267-015-0459-3 25813629

[B48] Phillips R. L. Wick A. F. Liebig M. A. West M. S. Daniels W. L. (2012). Biogenic emissions of CO2 and N2O at multiple depths increase exponentially during a simulated soil thaw for a northern prairie Mollisol. *Soil Biol. Biochem.* 45 14–22. 10.1016/j.soilbio.2011.09.012

[B49] Quast C. Pruesse E. Yilmaz P. Gerken J. Schweer T. Yarza P. (2013). The SILVA ribosomal RNA gene database project: improved data processing and web-based tools. *Nucleic Acids Res.* 41 D590–D596. 10.1093/nar/gks1219 23193283 PMC3531112

[B50] Ravishankara A. R. Daniel J. S. Portmann R. W. (2009). Nitrous oxide (N2O): the dominant ozone-depleting substance emitted in the 21st century. *Science* 326 123–125. 10.1126/science.1176985 19713491

[B51] Rochester I. J. (2003). Estimating nitrous oxide emissions from flood-irrigated alkaline grey clays. *Aust. J. Soil Res.* 41 197–206. 10.1071/SR02068

[B52] Roose-Amsaleg C. Yan C. Hoang A.-M. Laverman A. M. (2013). Chronic exposure of river sediments to environmentally relevant levels of tetracycline affects bacterial communities but not denitrification rates. *Ecotoxicology* 22 1467–1478. 10.1007/s10646-013-1133-2 24105062

[B53] Samad M. D. S. Biswas A. Bakken L. R. Clough T. J. de Klein C. A. M. Richards K. G. (2016). Phylogenetic and functional potential links pH and N2O emissions in pasture soils. *Sci. Rep.* 6:35990. 10.1038/srep35990 27782174 PMC5080606

[B54] Sanford R. A. Wagner D. D. Wu Q. Chee-Sanford J. C. Thomas S. H. Cruz-García C. (2012). Unexpected nondenitrifier nitrous oxide reductase gene diversity and abundance in soils. *Proc. Natl. Acad. Sci. U.S.A.* 109 19709–19714. 10.1073/pnas.1211238109 23150571 PMC3511753

[B55] Schloss P. D. Westcott S. L. Ryabin T. Hall J. R. Hartmann M. Hollister E. B. (2009). Introducing mothur: open-source, platform-independent, community-supported software for describing and comparing microbial communities. *Appl. Environ. Microbiol.* 75 7537–7541. 10.1128/AEM.01541-09 19801464 PMC2786419

[B56] Schnabel E. L. Jones A. L. (1999). Distribution of tetracycline resistance genes and transposons among phylloplane bacteria in Michigan apple orchards. *Appl. Environ. Microbiol.* 65 4898–4907. 10543801 10.1128/aem.65.11.4898-4907.1999 PMC91659

[B57] Sengeløv G. Agersø Y. Halling-Sørensen B. Baloda S. B. Andersen J. S. Jensen L. B. (2003). Bacterial antibiotic resistance levels in Danish farmland as a result of treatment with pig manure slurry. *Environ. Int.* 28 587–595. 10.1016/S0160-4120(02)00084-3 12504155

[B58] Shoun H. Fushinobu S. Jiang L. Kim S.-W. Wakagi T. (2012). Fungal denitrification and nitric oxide reductase cytochrome P450nor. *Philos. Trans. R. Soc. Lond. B Biol. Sci.* 367 1186–1194. 10.1098/rstb.2011.0335 22451104 PMC3306627

[B59] Stein L. Y. Klotz M. G. (2017). The nitrogen cycle. *Curr. Biol.* 26 R94–R98. 10.1016/j.cub.2015.12.021 26859274

[B60] Sun M. Ye M. Liu K. Schwab A. P. Liu M. Jiao J. (2017). Dynamic interplay between microbial denitrification and antibiotic resistance under enhanced anoxic denitrification condition in soil. *Environ. Pollut.* 222 583–591. 10.1016/j.envpol.2016.10.015 28082131

[B61] Thiele-Bruhn S. (2003). Pharmaceutical antibiotic compounds in soils - a review. *J. Plant Nutr. Soil Sci.* 166 145–167. 10.1002/jpln.200390023

[B62] Thiele-Bruhn S. (2005). Microbial inhibition by pharmaceutical antibiotics in different soils—dose-response relations determined with the iron(III) reduction test. *Environ. Toxicol. Chem.* 24 869–876. 10.1897/04-166R.1 15839561

[B63] Thiele-Bruhn S. Beck I. C. (2005). Effects of sulfonamide and tetracycline antibiotics on soil microbial activity and microbial biomass. *Chemosphere* 59 457–465. 10.1016/j.chemosphere.2005.01.023 15788168

[B64] Thomson A. J. Giannopoulos G. Pretty J. Baggs E. M. Richardson D. J. (2012). Biological sources and sinks of nitrous oxide and strategies to mitigate emissions. *Philos. Trans. R. Soc. London B Biol. Sci.* 367 1157–1168. 10.1098/rstb.2011.0415 22451101 PMC3306631

[B65] Underwood J. C. Harvey R. W. Metge D. W. Repert D. A. Baumgartner L. K. Smith R. L. (2011). Effects of the antimicrobial sulfamethoxazole on groundwater bacterial enrichment. *Environ. Sci. Technol.* 45 3096–3101. 10.1021/es103605e 21384910

[B66] USEPA (2013). *Literature Review of Contaminants in Livestock and Poultry Manure and Implications for Water Quality.* Washington, DC: United States Environmental Protection Agency.

[B67] Wagg C. Bender S. F. Widmer F. van der Heijden M. G. A. (2014). Soil biodiversity and soil community composition determine ecosystem multifunctionality. *Proc. Natl. Acad. Sci. U.S.A.* 111 5266–5270. 10.1073/pnas.1320054111 24639507 PMC3986181

[B68] Wei W. Isobe K. Shiratori Y. Nishizawa T. Ohte N. Otsuka S. (2014). N2O emission from cropland field soil through fungal denitrification after surface applications of organic fertilizer. *Soil Biol. Biochem.* 69 157–167. 10.1016/j.soilbio.2013.10.044

[B69] Westergaard K. Müller A. K. Christensen S. Bloem J. Sørensen S. J. (2001). Effects of tylosin as a disturbance on the soil microbial community. *Soil Biol. Biochem.* 33 2061–2071. 10.1016/S0038-0717(01)00134-1 11976785

[B70] White T. J. Bruns S. Lee S. Taylor J. (1990). “Amplification and direct sequencing of fungal ribosomal RNA genes for phylogenetics,” in *PCR Protocols: A Guide to Methods and Applications*, eds Innis M. A. Gelfand D. H., J. J. Sninsky, and T. J. White (London: Academic Press), 315–322.

[B71] Wu D. Chen G. Zhang X. Yang K. Xie B. (2017). Change in microbial community in landfill refuse contaminated with antibiotics facilitates denitrification more than the increase in ARG over long-term. *Sci. Rep.* 7:41230. 10.1038/srep41230 28120869 PMC5264584

[B72] Yin G. Liu M. Zheng Y. Li X. Lin X. Yin G. (2017). Effects of multiple antibiotics exposure on denitrification process in the Yangtze Estuary sediments. *Chemosphere* 171 118–125. 10.1016/j.chemosphere.2016.12.068 28012383

[B73] Zhu Y.-G. Johnson T. A. Su J.-Q. Qiao M. Guo G.-X. Stedtfeld R. D. (2013). Diverse and abundant antibiotic resistance genes in Chinese swine farms. *Proc. Natl. Acad. Sci. U.S.A.* 110 3435–3440. 10.1073/pnas.1222743110 23401528 PMC3587239

[B74] Zielezny Y. Groeneweg J. Vereecken H. Tappe W. (2006). Impact of sulfadiazine and chlorotetracycline on soil bacterial community structure and respiratory activity. *Soil Biol. Biochem.* 38 2372–2380. 10.1016/j.soilbio.2006.01.031

