# Supplementary figures and images for: Antibiotic Effects on Microbial Communities Responsible for Denitrification and N2O Production in Grassland Soils
Source: Front Microbiol. 2018 Sep 11;9:2121. doi: 10.3389/fmicb.2018.02121 (PMC6141661; doi:10.3389/fmicb.2018.02121)

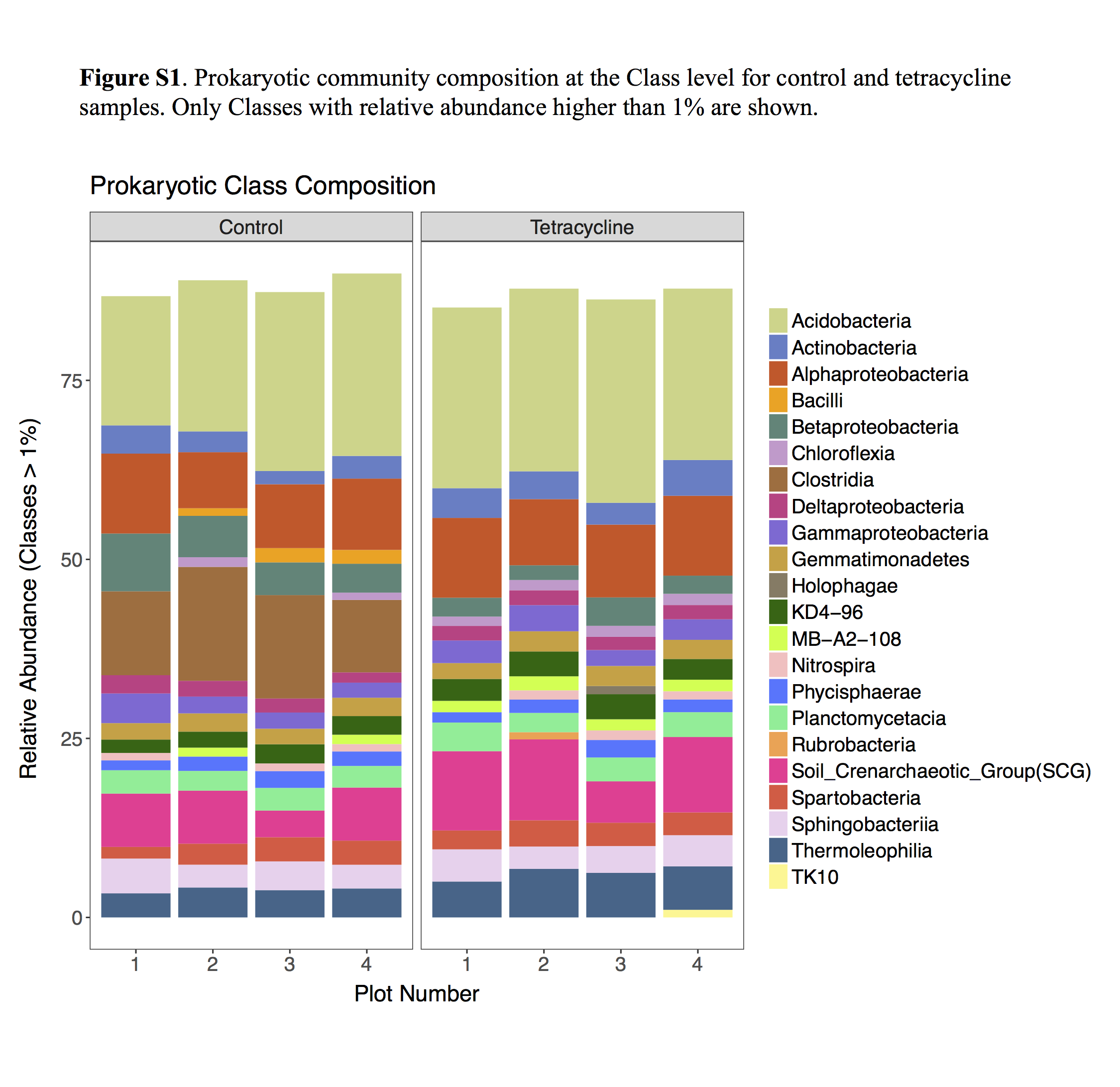

Supplement: Supplementary file 1 [file Image_1.TIFF]

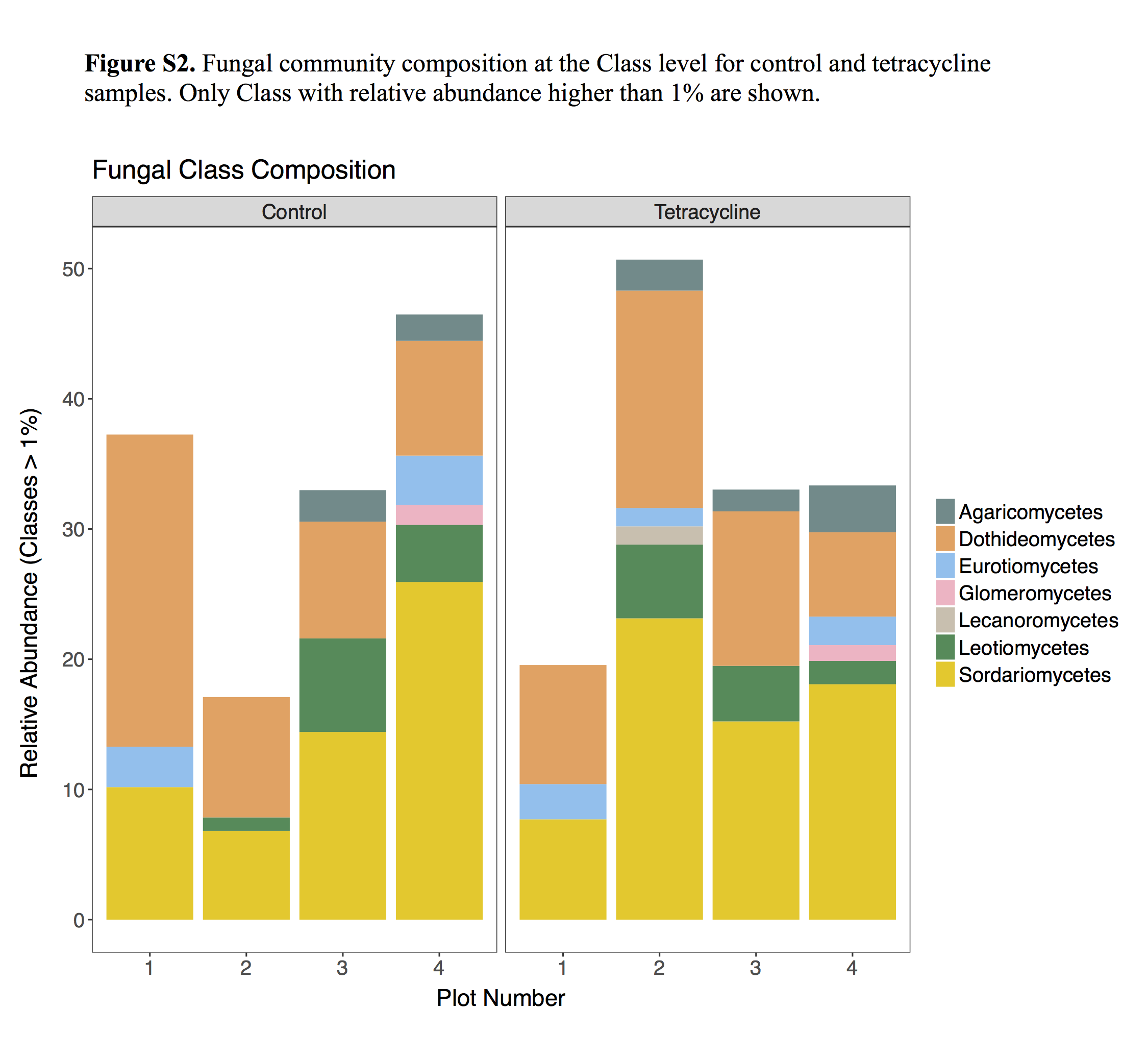

Supplement: Supplementary file 2 [file Image_2.TIFF]

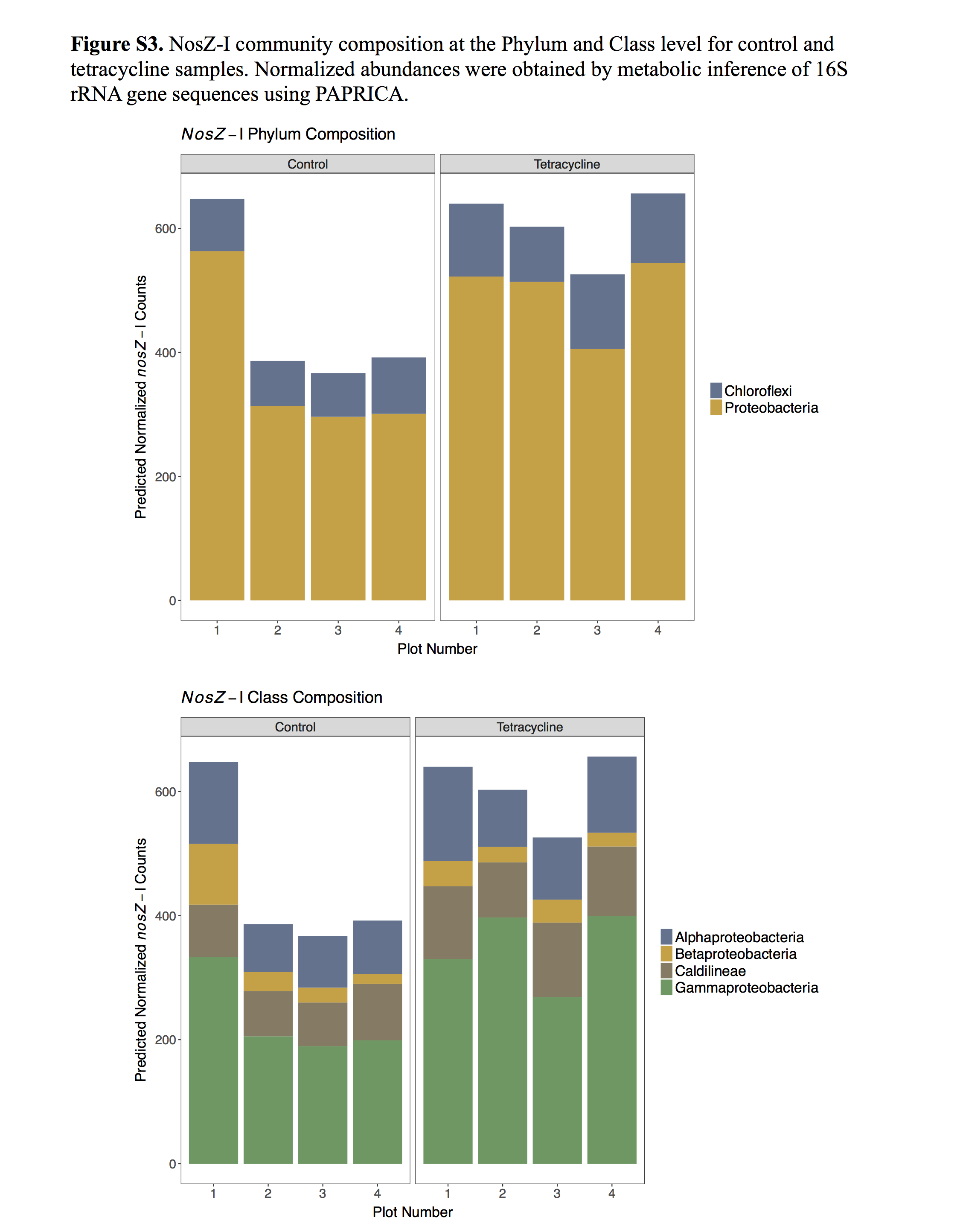

Supplement: Supplementary file 3 [file Image_3.TIFF]

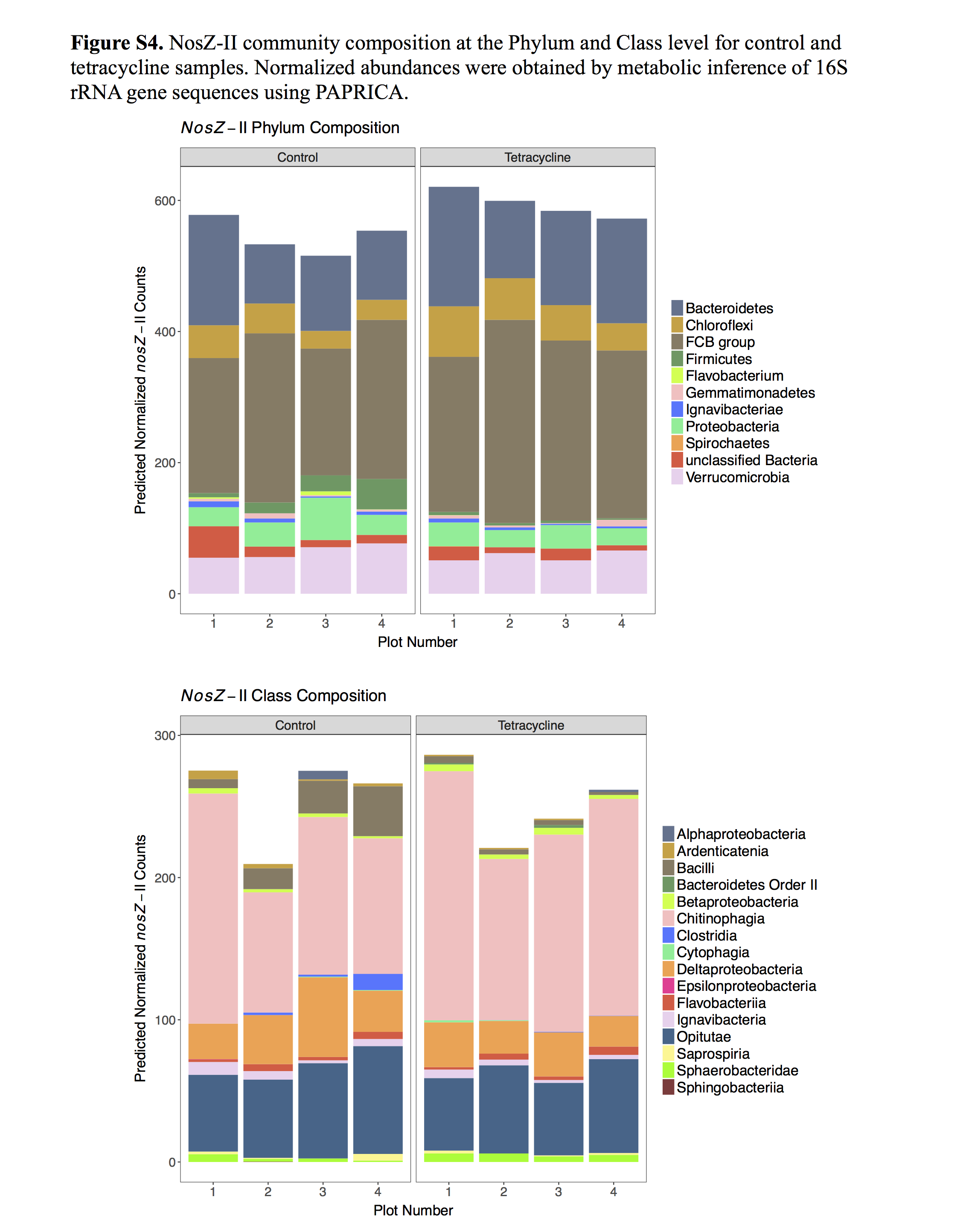

Supplement: Supplementary file 4 [file Image_4.TIFF]
